# Mechanisms of Extracellular Immunomodulation Mediated by Infectious Agents

**DOI:** 10.1155/2017/5107527

**Published:** 2017-05-10

**Authors:** Abel Viejo-Borbolla, Frank A. Schildberg, Hans-Gerhard Burgert

**Affiliations:** ^1^Institute of Virology, Hannover Medical School, 30625 Hannover, Germany; ^2^Department of Microbiology and Immunobiology, Harvard Medical School, Boston, MA 02115, USA; ^3^School of Life Sciences, University of Warwick, Coventry CV4 7AL, UK

Secreted proteins and proteins exposed on the cell surface, referred to here together as extracellular proteins (EPs), are essential in many biological processes, including development, homeostasis, inflammation, cancer, and pathogen-host interaction. EPs comprising both host and pathogen proteins are determinants of the outcome of infection. Therefore, it is of paramount importance to discover and further characterize these proteins and their interactions to better understand the infection process and pathogenesis and to develop novel therapeutic strategies.

In this “special issue,” we assembled a group of reviews and original research articles that deal with novel technologies for discovery of EPs involved in pathogen-host interaction, discuss the role of classical innate immune cytokines during viral infection, and devise novel therapeutic strategies using viral receptors as a target.

Despite the abundance and relevance of EPs, the identification and characterization of interactions with proteins of infectious agents is rather low when compared with that of intracellular proteins. Therefore, identifying and characterizing protein–protein interactions (PPI) between EPs from the host and infectious agents remain challenging and important tasks.

The recent development of novel technologies is facilitating this endeavor considerably. In her review, N. Martinez-Martin discusses classical and novel approaches to discover PPIs and explains their application to the identification of PPI between infectious agents and host proteins. Starting from biochemical and biophysical approaches, such as SPR and microarrays in various flavors, she discusses several mass spectrometry approaches. She also covers briefly genetic screens using cDNA libraries and more extensively the newer RNAi-based methods, CRISPR/Cas9 technology, and genetic screens with haploid cells. A valuable addition for the discovery of microbe-host PPIs appears to be the genomic variation in the pathogen and host population exposed to it, revealing positive selection in several cases. Multiple examples are given for successful identification of pathogen receptors or pathogen-host interactions with components of the immune system using these newer technologies. She also describes the inherent difficulties when applying current high throughput technologies to this task due to the biochemical nature of membrane proteins, causing problems with solubility, correct folding, and posttranslational modifications. Also, the fast dissociation rates and low affinity for some of these interactions require further modifications not present in the natural proteins. Considering these issues, it becomes clear that high throughput approaches based on soluble PPIs are an excellent screening platform but results must be confirmed by thorough specific experiments in relevant cells and/or animals.

One family of secreted proteins that plays key roles in virus-host interaction is that of cytokines, essential to coordinate the innate and adaptive immune responses. Due to their function, it is not surprising to find that infectious agents have devised many strategies to modulate their activities. These strategies include the expression of proteins with the ability to bind cytokines and/or modulate their interaction with the cytokine receptor, as well as the expression of transmembrane receptor and cytokine homologs. Among the cytokines, the interferons (IFN) are essential in protecting cells from viruses through the induction of an antiviral status. There are three types of IFN discovered to date: the multigene cytokine family of type I IFN containing IFN-*α* and *β* as prototypes, type II IFN including only IFN-*γ*, and type III IFN containing IFN-*λ*1–4. Two manuscripts deal with the interaction between IFN and virus infection in this special issue.

In the original research manuscript contributed by B. Hernáez et al., the authors study the effect of vaccinia virus (VACV) and its secreted, IFN-binding protein, B18, on the cellular type I IFN response. VACV is a member of the Poxviridae, large double stranded DNA viruses that encode many immunomodulatory proteins. In particular, they express secreted proteins with the ability to bind type I–III IFN [1–3]. VACV is one of the best-studied poxviruses in part due to its use as the vaccine employed during the world health organization-led campaign to eradicate smallpox, caused by the deadly variola virus. VACV expresses several genes whose protein products interfere with the IFN pathway and therefore can be used as a virus model to study evasion of IFN response. One of them, B18, is a secreted protein that binds type I IFN of different species with very high affinity, blocking the interaction between IFN and its receptor. Lack of B18 expression in VACV results in virus attenuation, indicating that it is a virulence factor [1]. By using a systems biology approach, the authors address whether B18 alone or in the context of VACV infection triggers changes in the global cellular transcriptome. They clearly show that the VACV B18 IFN-binding protein alone does not generally affect cellular gene expression and that replication competent VACV induces a global change in gene expression without enhancing that of IFN-related pathways. Similarly, they address whether addition of IFN has an effect on gene expression in the presence of VACV. Finally, they prove that VACV infection abrogated IFN-mediated antiviral response through both B18-dependent and independent mechanisms.

A different view on IFN is provided in the paper by J. Bruening et al. that reviews the current knowledge on IFN-*λ* expression and activity and its role during viral infection, focusing on hepatitis C virus (HCV). The authors describe the discovery and properties of IFN-*λ* and its receptor. IFN-*λ* signaling and induction of IFN-stimulated genes are, with some exceptions, quite similar to those of type I IFN. However, there are differences in the kinetics of activity and in the type of responsive tissue since expression of the IFN-*λ* receptor is restricted to epithelial cells. Most infectious agents, in particular viruses, infect epithelial cells at one or more stages of their life cycle. Therefore, the interaction between IFN-*λ* and pathogens may influence primary infection, spread, and pathogenicity. Focusing on the hepatotropic flavivirus HCV that can cause chronic infections leading to liver cirrhosis and hepatocellular carcinoma associated with high mortality, the authors describe HCV biology, pathogenesis, and current treatments. Newly developed antivirals targeting nonstructural proteins are highly successful in clearing HCV infection. However, due to their high cost and the fact that these do not inhibit reinfection, novel approaches are required. Finally, the authors review the innate immune response to HCV focusing on in vitro and in vivo studies addressing HCV-IFN-*λ* interaction. The authors discuss the interesting and initially counterintuitive data indicating that high levels of IFN-*λ* correlate with HCV chronicity of infection. Also, they explain the relationship between IFN-*λ* polymorphism, HCV spontaneous clearance, and the response to anti-HCV treatment.

The review article by J. M. Rojas et al. focuses on the anti-inflammatory master regulator IL-10 and its implications during viral infections. The main function of IL-10 is to limit immunopathology during inflammation, thereby protecting the host from tissue damage and loss of organ function. IL-10 is not only expressed by a variety of immune cells but is also able to regulate the activity of immune cells. It is, therefore, not surprising that viruses exploit this central immune regulatory pathway to evade immunity, leading to chronic/latent infections, and that detailed knowledge of the IL-10 effects is of high relevance. In this review, J. M. Rojas et al. discuss how the spatial and temporal delivery of IL-10 influences the course of viral infections. The focus of this review is factors that drive IL-10 expression during antiviral immune responses, cellular sources of IL-10, and IL-10-dependent regulatory mechanisms. These dynamic variables eventually impact how viruses can use this central regulatory pathway to undermine antiviral immune responses. The authors also discuss how a better understanding of both the basic expression level of IL-10 and effects of IL-10 on individual components of the immune system will enable the development of powerful immunotherapies against viral infections.

In another manuscript, K. Spiess et al. investigate a novel approach to treat human cytomegalovirus (HCMV), a highly prevalent beta herpesvirus that normally causes a primary asymptomatic infection. However, it causes disease associated with high morbidity and mortality in immunocompromised individuals, such as transplant recipients, and in the neonate. In this regard, HCMV is the leading infectious cause of congenital defects, above other pathogens like Zika virus. Therefore, development of novel therapeutic approaches to target HCMV is a public health priority worldwide.

In this study, K. Spiess et al. describe a therapeutic antiviral strategy against HCMV using immunotoxins, a protein-based therapy that takes advantage of known, specific ligand-receptor interactions. A typical immunotoxin contains a toxin linked to a targeting moiety to direct it to cells expressing the target receptor. Following binding to the receptor, the immunotoxin-receptor complex is internalized and the cell dies due to the action of the toxin. Several immunotoxins are currently being tested in clinical trials [4, 5]. The authors are pioneers in this field since they developed the first antiviral immunotoxin targeting the chemokine receptor US28 expressed by HCMV [6]. US28 is expressed at the plasma membrane of HCMV-infected cells and acts as a chemokine sink, binding and internalizing the ligands. Among the chemokines bound by US28 is CX3CL1, a chemokine that only interacts with one human chemokine receptor, CX3CR1. In this original research paper, the authors focus on CX3CL1-FTP, an immunotoxin containing a mutated form of CX3CL1 that specifically binds US28 over its cognate receptor CX3CR1 and that is linked to the catalytic domain of *Pseudomonas* endotoxin A. Based on the property that US28 binds to CX3CL1 with higher affinity than other chemokines and is internalized upon interaction [7], they explore novel strategies as attempts to systematically improve the selectivity and antiviral activity of this immunotoxin. This immunotoxin specifically kills HCMV-infected cells being a promising anti-HCMV tool. Finally, they discuss the possibility of using similar strategies to kill cells infected with the oncogenic herpesviruses, Kaposi's sarcoma-associated herpesvirus, and Epstein–Barr virus.

In conclusion, this special issue highlights different recent advances in immunomodulation mediated by infectious agents, focusing on novel regulatory strategies that implicate secreted factors or their receptors of both host and pathogen origin. This compilation of papers thereby gives an overview over novel technologies and discoveries to study EPs, which regulate a variety of biological processes and represent therapeutic targets of emerging importance.
